# Consumption of a dietary portfolio of cholesterol lowering foods improves blood lipids without affecting concentrations of fat soluble compounds

**DOI:** 10.1186/1475-2891-13-101

**Published:** 2014-10-18

**Authors:** Vanu R Ramprasath, David JA Jenkins, Benoit Lamarche, Cyril WC Kendall, Dorothea Faulkner, Luba Cermakova, Patrick Couture, Chris Ireland, Shahad Abdulnour, Darshna Patel, Balachandran Bashyam, Korbua Srichaikul, Russell J de Souza, Edward Vidgen, Robert G Josse, Lawrence A Leiter, Philip W Connelly, Jiri Frohlich, Peter JH Jones

**Affiliations:** Richardson Centre for Functional Foods and Nutraceuticals, Winnipeg, MB R3T 2 N2 Canada; Department of Human Nutritional Sciences, University of Manitoba, Winnipeg, MB Canada; Clinical Nutrition & Risk Factor Modification Center, Toronto, ON Canada; Department of Medicine, Division of Endocrinology and Metabolism, St. Michael’s Hospital, Toronto, ON Canada; Departments of Nutritional Sciences, University of Toronto, Toronto, ON Canada; Faculty of Medicine, University of Toronto, Toronto, ON Canada; Institute of Nutrition and Functional Foods, Laval University, Quebec City, Quebec; >Department of Pathology and Laboratory Medicine, University of British Columbia, Vancouver, British Columbia USA; Institute of Medical Science, Faculty of Medicine, University of Toronto, Toronto, ON Canada; Department of Clinical Epidemiology & Biostatistics, McMaster University, Hamilton, ON Canada; Keenan Research Centre of the Li Ka Shing Knowledge Institute, St. Michael’s Hospital, Toronto, ON Canada

**Keywords:** Plant sterols, Fat soluble vitamins, Portfolio diet

## Abstract

**Background:**

Consumption of a cholesterol lowering dietary portfolio including plant sterols (PS), viscous fibre, soy proteins and nuts for 6 months improves blood lipid profile. Plant sterols reduce blood cholesterol by inhibiting intestinal cholesterol absorption and concerns have been raised whether PS consumption reduces fat soluble vitamin absorption.

**Objective:**

The objective was to determine effects of consumption of a cholesterol lowering dietary portfolio on circulating concentrations of PS and fat soluble vitamins.

**Methods:**

Using a parallel design study, 351 hyperlipidemic participants from 4 centres across Canada were randomized to 1 of 3 groups. Participants followed dietary advice with control or portfolio diet. Participants on routine and intensive portfolio involved 2 and 7 clinic visits, respectively, over 6 months.

**Results:**

No changes in plasma concentrations of α and γ tocopherol, lutein, lycopene and retinol, but decreased β-carotene concentrations were observed with intensive (week 12:p = 0.045; week 24:p = 0.039) and routine (week 12:p = 0.031; week 24:p = 0.078) portfolio groups compared to control. However, cholesterol adjusted β-carotene and fat soluble compound concentrations were not different compared to control. Plasma PS concentrations were increased with intensive (campesterol:p = 0.012; β-sitosterol:p = 0.035) and routine (campesterol: p = 0.034; β-sitosterol: p = 0.080) portfolio groups compared to control. Plasma cholesterol-adjusted campesterol and β-sitosterol concentrations were negatively correlated (p < 0.001) with total and LDL-C levels.

**Conclusion:**

Results demonstrate that consuming a portfolio diet reduces serum total and LDL-C levels while increasing PS values, without altering fat soluble compounds concentrations. The extent of increments of PS with the current study are not deleterious and also maintaining optimum levels of fat soluble vitamins are of paramount necessity to maintain overall metabolism and health. Results indicate portfolio diet as one of the best options for CVD risk reduction.

**Trial registration:**

clinicaltrials.gov Identifier: NCT00438425

## Background

Randomized control trials using metabolically controlled designs [1–3] as well as effectiveness trials with free living designs [4, 5] have demonstrated reductions in blood LDL-C concentrations with consumption of a portfolio diet including plant sterols (PS), soy proteins, viscous fibres and nuts. Consumption of portfolio diet for 1 year under real world conditions by 66 participants resulted in a 13% reduction in LDL-C levels [5]. Similarly, a recently conducted study with portfolio diet for 6 months using a multicentre design across Canada showed a 13.8% reduction in LDL-C in 330 participants [4].

Plant sterols are one of the components of the portfolio diet known for their plasma cholesterol reducing property. Consumption of 2 g/d of PS reduces LDL-C by 13 to 16% [6]. PS are found to be effectively beneficial when combined with different dietary components such as fish oil, ascorbic acid, β-glucan and psyllium [7–9] as well as with statin drugs [10, 11]. Consumption of PS might not cause any major health concern [12], although some studies have implicated increased PS concentrations in plasma as being associated with elevated cardiovascular disease (CVD) risk [13–17]. Other recent studies with different dosages and study designs have not demonstrated that circulatory PS levels associate with risk of CVD and hence, consumption of portfolio diet containing PS may be safe and reduce risk of CVD [18–22].

Cholesterol lowering effects induced by the portfolio diet could occur through mechanisms including reduced intestinal cholesterol absorption due to PS [23], decreased cholesterol synthesis and elevated LDL-C receptor uptake of cholesterol in liver by soy proteins [24] increased bile acid loss by the action of dietary fibre [25] and almonds are sources of vegetable proteins, monounsaturated fats and PS and are likely to produce their effects by a range of mechanisms [26]. As PS act mainly by inhibiting intestinal cholesterol absorption, it might be possible that the absorption of other fat soluble compounds such as carotenoids, tocopherols and retinoids are compromised by the PS containing portfolio diet consumption [27]. Vitamin A is essential for multiple functions in the human body including maintenance of cell function, growth, vision, epithelial integrity, immune function, and reproduction. Deficiency of vitamin A leads to vision loss, increased morbidity, and mortality [28, 29]. Tocopherols are strong antioxidants and play important role in prevention of chronic diseases associated with oxidative stress such as cardiovascular disease, atherosclerosis, and cancer [30]. Deficiency of vitamin E leads to anemia and in addition severe deficiency could result in neuromuscular abnormalities characterized by spinocerebellar ataxia and myopathies [30–32].

A few studies have raised concerns about reduction in the absorption of fat soluble vitamins and carotenoids including β-carotenes and α-tocopherols following PS intake [33–38]. In contrast, other interventions have shown no changes in plasma fat soluble vitamin concentrations with PS consumption [39–42]. Although combination of PS with other portfolio dietary components showed significant reductions in plasma LDL-C concentrations, effects of portfolio ingredients on plasma fat soluble vitamins levels have never been studied. Hence our aim was to determine whether the portfolio diet with the presence of PS affects the plasma fat soluble vitamin concentrations in hypercholesterolemic participants. Additional objectives were to determine the extent of the increment in plasma PS concentration following a portfolio diet and determine the correlations with other lipid markers in blood.

### Participants and methods

#### Participants

We have previously reported the results on the effect of portfolio diet on CVD risk factors including plasma LDL-C levels [4]. Detailed information on the study design and participant characteristics of this multi-centre clinical study has been provided [4]. Briefly, 351 hyperlipidemic participants (137 males and 214 postmenopausal females) were randomized for the study at 4 different centers across Canada including Quebec City, Toronto, Winnipeg and Vancouver. The main inclusion criteria were males and post-menopausal females with low to intermediate Framingham 10-year risk categories with 3.50-5.31 and 3.00-4.61 mmol/L LDL-C respectively. The following were considered as exclusion criteria; history of cardiovascular disease, cancer or strong family history of cancer, untreated hypertension with blood pressure >140/90 mmHg, diabetes, hepatic or renal disease and currently under lipid lowering therapy. The trial is registered in clinical trials.gov registry (Identifier: NCT00438425).

### Study protocol

**Figure 1:**
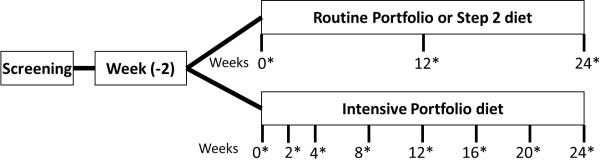
**Schematic representation of the study protocol.** *Indicates visit to the centre; collection of 7-day diet record/check lists, satiety score; body weight and blood pressure measurement and fasting blood collection.

Participants were recruited by advertisements. The study protocol was explained to all participants and signatures on informed consent forms were obtained from eligible participants. Eligible participants were randomized in blocks of 75 participants with stratification by centre, gender and baseline LDL-C concentrations with greater than or lesser than 4.09 mmol/L. In this study with a parallel design, participants were randomized to one of the three treatment groups with either a therapeutic low fat diet control or dietary portfolio of cholesterol lowering foods with either 2 visits (routine) or 7 visits (intensive) during a 6 month period (Figure 1). Participants visited the respective centers at baseline and by 3 and 6 months for the control and portfolio routine diet groups. Participants categorized to intensive dietary portfolio group visited the respective centers at baseline, 2 weeks and subsequently every month. During each of the visits, the preceding 7-day dietary histories were collected and reviewed along with recording the body weight and blood pressure. Fasting blood samples were collected from the participants during each visit. Serum lipid profile and apolipoproteins were measured as explained earlier [4]. This study was conducted according to the guidelines laid down in the Declaration of Helsinki and all procedures were approved by the ethics committees of Universities of British Columbia, Laval, Manitoba and Toronto as well as St Michael’s Hospital, Toronto.

Diets consumed by the participants were previously explained in detail [4]. During the 6 month study treatment period, participants received dietary advice by dieticians to follow a weight maintaining vegetarian diets with foods available at supermarkets and health food stores. Participants in the dietary portfolio groups were recommended to incorporate the portfolio dietary study foods into their diets. The aim was to provide 0.94 g of PS in margarine, 9.8 g of viscous fibre, 22.5 g of soy proteins and 22.5 g of nuts per 1000 kcal of diet per day. Control dietary participants were advised to consume low fat dairy and whole grain cereals along with fruits and vegetables and to avoid the portfolio dietary components. All participants were supplied with measuring cups and spoons to control the amount of portfolio and control dietary foods consumption. Diets were analyzed using a program based on US Department of Agriculture data (ESHA Food Processor SQL version 10.1.1; ESHA, Salem, Oregon).

Concentrations of plasma α and γ tocopherols, β-carotene, lutein, lycopene and retinol were measured concurrently with an isocratic high performance liquid chromatograph (HPLC) (1100 HPLC, Agilent Technologies, Palo Alto, California) as described earlier [43]. Briefly, internal standards retinol acetate and β-apo-8′-carotenal in methanol were added to each of the samples and deproteinized with ethanol followed by extraction with hexane. Samples were injected into a C18 reverse phase column (Zorbax Eclipse XBD, Agilent Technologies, Palo Alto, California) with a guard column and eluted with mobile phase consisting of methanol, acetonitrile and tetrahydrofuran in the ratio of 75:20:5 (v/v/v). Detection wavelengths were set at 290, 320 and 450 nm for detection of the compounds of interest. Fat-soluble vitamins were identified using authentic standards (Sigma-Aldrich) and were quantified using standard curves.

Concentrations of PS in plasma were measured by gas chromatography (6890 GC, Agilent Technologies, Palo Alto, California) equipped with a flame ionization detector and auto-injector system. A 30-m SAC-5 column (Sigma-Aldrich Canada Ltd., Oakville, Ont.) was used. Briefly, 5-α cholestane as an internal standard was added to each of the samples followed by addition of methanolic potassium hydroxide and saponification. Sterols were extracted from the mixture with petroleum ether. Extracted samples were derivatized with TMS reagent (pyridine-hexamethyldisilazane-trimethylchlorosilane (9:3:1, v/v)) and samples were injected into the GC [44]. The injector and detector were set at 300 and 310 degrees C, respectively. The flow rate of the carrier gas, helium was 1.2 ml/min with the inlet splitter set at 100:1. Individual PS were identified using authentic standards (Sigma-Aldrich Canada Ltd., Oakville, Ont). Internal standards were used to calculate detector response factors. Campesterol and β-sitosterol concentrations were determined by identifying the peak sizes and expressing them relative to 5-α cholestane internal standard. As PS and fat soluble vitamins are mainly transported in cholesterol-containing particles in serum, the absolute concentrations were adjusted for the serum cholesterol concentration to eliminate the effect of changes in the serum cholesterol concentration.

### Statistical analyses

Data are expressed as means with their standard errors. The significance of the differences between week 0, week 12 as well as week 24 between different groups were assessed by Least Squares Means utilizing PROC MIXED procedure in SAS, with Tukey adjustment for multiplicity of comparisons. Analysis of covariance was performed with sex, treatment, centre and centre-by-treatment interaction as main effects and baseline as a covariate. In all tests of hypotheses, p values <0.05 were considered significant. All statistical analyses were performed using the SAS software (version 9.2; SAS Institute Inc., Cary, NC, USA) Sample size determination with LDL-C as primary end point has been explained in detail earlier [4].

## Results

### Baseline characteristics of participants and dietary intakes during the study

Baseline characteristics of participants of all treatments were similar except for the relatively higher ratio of men to women on the intensive portfolio group compared to the other groups (Table 1). Adherence to the dietary recommendations assessed from the 7 day food records using food processor software was 46.4% and 40.6% for the intensive and routine portfolio diets, respectively [4]. Dietary macronutrient profile of the portfolio and control diets were explained in Table 2. Participants in intensive and routine portfolio groups consumed 0.8 and 0.6 g/d/1000 Kcal PS in their diets, respectively. The amount of vitamin A consumed during the study by participants in intensive and routine portfolio dietary groups was 1280 and 1248 IU compared with control group which was 1699 IU per day. In contrast, intakes of vitamin E were higher with participants in intensive (3.0 IU/d) and routine (2.8 IU/d) portfolio groups compared to the control dietary group (1.6 IU/d).

**Table 1:** **Baseline characteristics of participants**

Characteristic	Treatments			P-value ^***a, b***^
	Portfolio intensive (n = 101)	Portfolio routine (n = 122)	Control (n = 122)	
Age, mean (SD) y	54.6 (9.8)	57.1 (8.3)	56.9 (9.3)	0.082
Sex				
Male	50 (49.5%)	37 (30.3%)	47 (38.5%)	0.014
Female	51 (50.5%)	85 (69.7%)	75 (61.5%)	
Body weight, mean (SD), kg	75.5 (13.5)	73.7 (13.3)	76.9 (13.8)	0.189
Body mass index, mean (SD), kg/m^2^	26.6 (4.0)	26.9 (3.8)	27.4 (3.9)	0.244
Blood pressure, mean (SD), mm Hg				
Systolic	120.9 (12.6)	119.5 (13.5)	119.9 (11.9)	0.683
Diastolic	73.1 (9.0)	73.8 (8.3)	73.0 (8.0)	0.731
Lipids, mean (SD), mmol/L^*c*^				
Total	6.53 (1.00)	6.63 (1.06)	6.45 (0.87)	0.830
LDL-C	4.42 (0.89)	4.50 (0.88)	4.35 (0.72)	0.380
HDL-C	1.42 (0.32)	1.40 (0.40)	1.39 (0.36)	0.836
Triglycerides	1.53 (0.73)	1.61 (0.83)	1.65 (0.98)	0.574
Medication use				
Lipid lowering medication	13 (12.9%)	20 (16.4%)	18 (14.8%)	0.754
Antihypertensive medication	18 (17.8%)	17 (13.9%)	28 (23.0%)	0.193
Hormone-replacement medication	2 (2%)	7 (5.7%)	2 (1.6%)	0.195
Thyroxine	9 (8.9%)	11 (9%)	15 (12.3%)	0.627

^*a*^p-values calculated by ANOVA for continuous variables.

^*b*^p-values calculated by CHI2/Fisher’s Exact Test for categorical variables.

^*c*^Mean lipid levels for 100 subjects in the portfolio intensive group, for 122 subjects in the portfolio routine, and for 121 subjects in the control.

**Table 2:** **Macronutrient intake profiles of portfolio and control diets during the study**

Nutrients	Portfolio intensive (n = 101)	Portfolio routine (n = 122)	Control (n = 122)
Energy, Kcal/d	1977	1804	1802
Total protein	88(18)	79(18)	80(18)
Available carbohydrate	225(45)	208(46)	225(50)
Total dietary fibre (g/100 kcal)	42(22)	36(20)	31(17)
Total fat	70(32)	63(31)	52(26)
Saturated fatty acid	14(7)	14(7)	14(7)
Monounsaturated fatty acid	27(12)	24(12)	19(9)
Polyunsaturated fatty acid	18(9)	16(8)	11(6)
Cholesterol (mg/1000 kcal)	123(66)	120(68)	154(86)

Data are expressed as mean grams per day (% energy intake) unless stated and based on the dietary data obtained at week 24 of the study.

### Serum lipid profile changes with portfolio and control diets

Reductions in serum total and LDL-C level with consumption of portfolio diets are reported in Table 3 [4]. No differences were found between the 3 intervention groups in baseline blood lipid concentrations. Percentage change and absolute treatment differences between the control and the two dietary portfolio interventions were significant for LDL-C (p < 0.001) and in absolute units for the TC:HDL-C ratio (intensive dietary portfolio: p = 0.004, routine dietary portfolio: p = 0.006), with no significant differences seen between the two dietary portfolio groups (p = 0.66).

**Table 3:** **Effect of portfolio and control diets on blood lipids levels and the differences between dietary interventions**

	Treatments ^***a***^						Between treatment differences ^***b***^					
Variable	Portfolio intensive (n = 101)		Portfolio routine (n = 122)		Control (n = 122)		PI vs. PR		PI vs. C		PR vs. C	
	Wk 0	Wk 24	Wk 0	Wk 24	Wk 0	Wk 24	Mean (95% CI)	P-value	Mean (95% CI)	P-value	Mean (95% CI)	P-value
Cholesterol, mmol/L												
Total	6.53	5.88	6.63	6.04	6.46	6.41	-0.07 (-0.31, 0.17)	0.777	-0.56 (-0.80, -0.33)	<0.0001	-0.49 (-0.73, -0.26)	<0.0001
LDL-C	4.42	3.80	4.50	3.92	4.35	4.24	-0.06 (-0.27, 0.15)	0.773	-0.46 (-0.67, -0.26)	<0.0001	-0.40 (-0.60, -0.20)	<0.0001
HDL-C	1.42	1.39	1.40	1.39	1.39	1.40	0.007 (-0.006, 0.072)	0.965	-0.02 (-0.09, 0.04)	0.619	-0.03 (-0.09, 0.03)	0.453
Triglycerides	1.52	1.50	1.61	1.60	1.66	1.72	-0.06 (-0.23, 0.12)	0.743	-0.10 (-0.27, 0.07)	0.372	-0.04 (-0.21, 0.13)	0.824
Total:HDL-C	4.81	4.44	5.01	4.60	4.93	4.87	-0.05 (-0.27, 0.17)	0.852	-0.33 (-0.54, -0.11)	0.001	-0.28 (-0.50, -0.06)	0.008
Apolipoproteins g/L												
A1	1.60	1.60	1.60	1.60	1.60	1.59	0.02 (-0.02, 0.06)	0.533	0.01 (-0.03, 0.05)	0.868	-0.01 (-0.05, 0.03)	0.819
B	1.23	1.10	1.26	1.14	1.20	1.20	-0.01 (-0.06, 0.03)	0.846	-0.11 (-0.16, -0.07)	<0.0001	-0.10 (-0.15, -0.06)	<0.0001

^*a*^For paired samples *T*-test of change from baseline, done on absolute change values.

^*b*^Using analysis of covariance with sex, treatment and sex by treatment interaction as main effects and baseline as a covariate. A Tukey adjustment was made for multiple comparisons. *Abbreviations*: PI Portfolio intensive, PR Portfolio routine, C Control.

### Effect of portfolio diet on plasma fat soluble compound concentrations

Plasma concentrations of tocopherols, carotenoids and retinol and their ratios to cholesterol are shown in Table 4. Participants consuming the intensive portfolio diet showed no changes in any of the fat soluble vitamins measured except for a slight but significant increase in cholesterol adjusted α-tocopherol (p = 0.014) and retinol (p = 0.032) at week 12, but not at week 24. Furthermore, concentrations of β-carotene and retinol were found to be decreased with intensive portfolio diet only at week 24 (β-carotene: p = 0.017; retinol: p = 0.007). No significant changes in any fat soluble vitamin concentrations were noted in routine portfolio diet group, except for an increase in cholesterol adjusted γ-tocopherol at week 12 (p = 0.003), as well as at week 24 (p = 0.014) and decreased β-carotene (p = 0.016) and α-tocopherol (p = 0.041) only at week 12, but not at week 24. Consumption of the control diet had no impact on plasma vitamin concentrations except for an increase in cholesterol adjusted β-carotene at week 12 (p = 0.018) but not at week 24.

**Table 4:** **Plasma concentrations of carotenoids, tocopherols and retinoids following portfolio and control diets**

Variable	Intensive portfolio						Routine portfolio						Control					
	Wk 0		Wk 12 ^***a***^		Wk 24 ^***a***^		Wk 0		Wk 12 ^***a***^		Wk 24 ^***a***^		Wk 0		Wk 12 ^***a***^		Wk 24 ^***a***^	
	Mean	SEM	Mean	SEM	Mean	SEM	Mean	SEM	Mean	SEM	Mean	SEM	Mean	SEM	Mean	SEM	Mean	SEM
**α-tocopherol** ^***b***^	47.72	2.51	45.60	2.71	45.23*	2.55	50.15	2.37	47.36*	2.77	47.50	2.83	51.05	2.71	51.76	2.99	49.72	3.05
**γ-tocopherol** ^***b***^	4.97	0.22	4.76	0.29	4.71	0.29	5.28	0.30	5.10	0.35	4.99	0.36	4.96	0.30	5.22	0.37	5.21	0.35
**Lycopene** ^***b***^	2.61	0.13	2.49	0.23	2.49	0.13	2.78	0.20	2.53	0.17	2.56	0.17	3.21	0.20	3.23	0.17	3.06	0.23
**Lutein** ^***b***^	1.65	0.19	1.51	0.21	1.64	0.21	1.80	0.17	1.67	0.16	1.80	0.18	1.71	0.15	1.77	0.18	1.69	0.19
**β-carotene** ^***b***^	2.24	0.19	2.07^†^	0.18	2.06*^†^	0.19	2.27	0.17	2.11*^†^	0.17	2.20^†^	0.17	2.23	0.18	2.38	0.18	2.45	0.17
**Retinol** ^***b***^	2.78	0.12	2.65	0.16	2.65*	0.15	2.94	0.15	2.88	0.13	2.83	0.15	2.73	0.12	2.86	0.15	2.84	0.19
**α-tocopherol:TC** ^***c***^	7.46	0.46	8.39*	0.51	8.00	0.49	7.91	0.46	8.41	0.55	8.25	0.49	8.07	0.53	8.54	0.53	8.04	0.51
**γ-tocopherol:TC** ^***c***^	0.79	0.04	0.88	0.05	0.81	0.05	0.80	0.05	0.91*	0.07	0.88*	0.07	0.81	0.06	0.86	0.06	0.83	0.05
**Lycopene:TC** ^***c***^	0.42	0.02	0.45	0.04	0.44	0.02	0.42	0.04	0.44	0.03	0.45	0.03	0.50	0.04	0.54	0.03	0.49	0.04
**Lutein:TC** ^***c***^	0.29	0.04	0.29	0.04	0.30	0.04	0.29	0.03	0.31	0.03	0.32	0.03	0.28	0.03	0.29	0.03	0.28	0.03
**β-carotene:TC** ^***c***^	0.35	0.04	0.38	0.03	0.37	0.04	0.36	0.03	0.38	0.03	0.38	0.03	0.35	0.03	0.40*	0.03	0.39	0.03
**Retinol:TC** ^***c***^	0.43	0.02	0.49*	0.03	0.47	0.03	0.47	0.03	0.52	0.03	0.50	0.03	0.43	0.02	0.47	0.03	0.46	0.03

*Indicates significance compared to respective baseline. ^†^indicates significance with changes from baseline compared to control at respective time point.

^a^Using analysis of covariance with sex, treatment and sex by treatment interaction as main effects and baseline as a covariate. A Tukey adjustment was made for multiple comparisons. ^*b*^μmol/L. ^***c***^μmol/L:mmol/L.

No differences between the three dietary interventions were observed with changes from respective baselines of any of fat soluble vitamins measured except for a significant reduction in concentration of β-carotene at week 12 with intensive (p = 0.045) and routine (p = 0.031) portfolio diet, compared to changes with control diet. However, the change from baseline in β-carotene concentration at week 24 with routine portfolio diet group was not different (p = 0.078) when compared to change with control diet group, but was lower with >intensive portfolio diet (p = 0.039) compared to changes from baselines observed in control dietary group. Furthermore, cholesterol adjusted concentrations of fat soluble vitamins, especially β-carotene, were not different across intervention groups. Changes in cholesterol adjusted concentrations of plasma fat soluble compounds with portfolio diet consumption were negatively correlated with changes in total cholesterol (α-tocopherol: p = 0.005; retinol: p = 0.008; lutein: p = 0.031; lycopene: p = 0.004; β-carotene: p = 0.009) and LDL-C (α-tocopherol: p = 0.004; retinol: p = 0.004; lutein: p = 0.027; lycopene: p = 0.004; β-carotene: p = 0.013) levels (Table 5). Furthermore, changes in cholesterol adjusted plasma concentrations of α-tocopherol (p = 0.026), retinol (p = 0.038) and lycopene (p = 0.048) showed a negative correlation with serum apoB concentrations.

**Table 5:** **Correlations between changes in plasma carotenoid, tocopherol and retinoid concentrations with lipid markers after consumption of portfolio**

Variable		γ-tocopherol:TC	α-tocopherol:TC	Retinol:TC	Lutein:TC	Lycopene:TC	β-carotene:TC
**TC**	N	161	161	161	159	157	156
	r	-0.111	-0.219	-0.209	-0.171	-0.230	-0.209
	P	0.161	0.005	0.008	0.031	0.004	0.009
**LDL-C**	N	160	160	160	158	156	155
	r	-0.088	-0.225	-0.228	-0.176	-0.229	-0.200
	P	0.271	0.004	0.004	0.027	0.004	0.013
**ApoB**	N	161	161	161	159	157	156
	r	-0.073	-0.175	-0.163	-0.138	-0.158	-0.129
	P	0.360	0.026	0.038	0.083	0.048	0.110

### Changes in plasma plant sterol concentrations during the study

**Figure 2:**
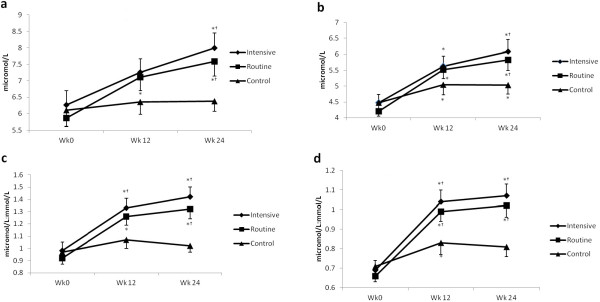
**Plasma concentrations of plant sterols and their ratios with cholesterol after consumption of portfolio and control diets.** **a**. Campesterol, **b**. β-sitosterol, **c**. campesterol:TC, **d**. β-sitosterol:TC. *Indicates significance compared to respective baseline. ^†^indicates significance with changes from baseline compared to control at respective time point. ^*b*^indicates significance with changes from baseline compared to routine portfolio at respective time point. Using analysis of covariance with sex, treatment and sex by treatment interaction as main effects and baseline as a covariate. A Tukey adjustment was made for multiple comparisons.

Plasma PS concentrations including campesterol and β-sitosterol and their ratio with cholesterol are depicted in Figure 2. The intensive portfolio diet group showed an increase in concentration of campesterol at week 24 (p = 0.034) compared with baseline. Similarly, participants consuming the routine portfolio dietary group also demonstrated increased campesterol concentrations at both weeks 12 (p < 0.001) and 24 (p < 0.001), respectively, compared to baseline. Ratios of campesterol and cholesterol were also found to be increased by intensive (p = 0.001 at week 12, p < 0.001at week 24) as well as routine (p < 0.001 at week 12 and 24) portfolio diet consumption. No significant changes in campesterol and cholesterol-adjusted campesterol concentrations were observed after consuming the control diet. Plasma concentrations of β-sitosterol were found to be increased in both intensive (p = 0.008 at week 12, p < 0.001 at week 24) and routine (p < 0.001 at week 12 and 24) portfolio diet groups. Increases in cholesterol adjusted β-sitosterol concentrations in plasma were similarly elevated as unadjusted β-sitosterol concentrations (p < 0.001) at week 12 and 24 after intensive or routine portfolio diet consumptions compared to their baselines. Concentrations of β-sitosterol and its ratio to cholesterol were significantly elevated in participants consuming control diet at 12 weeks (β-sitosterol: p = 0.024; ratio to cholesterol p = 0.032) as well as at 24 weeks (p = 0.05).

No differences across time were observed across participants consuming intensive and routine portfolio diet in circulating campesterol and β-sitosterol levels, or their ratios to cholesterol. However, increased plasma campesterol and β-sitosterol concentrations were observed with both intensive (week 24: p = 0.012 for campesterol and p = 0.035 for β-sitosterol) as well as routine (week 24: p = 0.034 for campesterol and p = 0.080 for β-sitosterol) portfolio diet groups, compared with control diet. Plasma campesterol and β-sitosterol concentrations after adjusting for blood cholesterol concentrations were also found to be elevated after consumption of intensive (week 12: p = 0.037 for campesterol:TC and p = 0.022 for β-sitosterol:TC; week 24: p = 0.001 for campesterol:TC and p = 0.002 for β-sitosterol:TC) and routine (week 12: p = 0.054 for β-sitosterol:TC; week 24: p = 0.003 for campesterol:TC and p = 0.002 for β-sitosterol:TC) portfolio diets, compared with consumption of the control diet.

Changes in plasma campesterol:TC ratio with portfolio diet were found to be negatively correlated with total (p = 0.0001) and LDL-C (p = 0.0004), as well as with apoA1 (p = 0.001) and ApoB (p = 0.002) levels and LDL-C/ApoB ratio (p = 0.009) (Table 6). Similarly, changes in plasma β-sitosterol:TC ratios after consuming portfolio diets were also observed to be negatively correlated with the change in total (p = 0.0002) and LDL-C (p = 0.0001) and ApoB (p = 0.0004) levels, as well as in cholesterol:HDL-C ratio (p = 0.021), LDL-C:HDL-C ratio (p = 0.010), apoB: apoA1 ratio (p = 0.001) and LDL-C:apoB ratio (p = 0.036).

**Table 6:** **Correlations between changes in plasma plant sterol concentrations and lipid markers with portfolio diet consumption**

Variable		Campesterol:TC	β-sitosterol:TC
**TC**	N	156	152
	R	-0.302	-0.294
	P	0.0001	0.0002
**LDL-C**	N	155	151
	R	-0.281	-0.307
	P	0.0004	0.0001
**ApoA1**	N	156	152
	R	-0.269	-0.019
	P	0.001	0.815
**ApoB**	N	156	152
	R	-0.242	-0.285
	P	0.002	0.0004
**TC/HDL-C**	N	155	151
	R	-0.008	-0.188
	P	0.921	0.021
**LDL-C/HDL-C**	N	155	151
	R	-0.046	-0.209
	P	0.568	0.010
**ApoB/ApoA1**	N	156	152
	R	-0.056	-0.265
	P	0.488	0.001
**LDL-C/ApoB**	N	154	150
	R	-0.211	-0.172
	P	0.009	0.036

## Discussion

Results demonstrate that intake of a portfolio diet for 6 months improved blood lipid concentrations without altering the plasma fat soluble vitamin concentrations after adjustment for cholesterol levels. Phytosterol intake has been believed to result in a possible reduction in absorption of fat soluble vitamins which in turn might lead to their lower levels in plasma [33, 34, 37]. Carotenoids, retinols and tocopherols are absorbed in the intestine in a similar fashion to lipids. PS lower blood cholesterol by inhibiting cholesterol absorption in the gut. Hence, the possibility exists that consumption of PS might reduce circulating concentrations of these fat soluble compounds due to their reduced intestinal absorption [27]. Reductions in serum carotenoids and lycopene concentrations in normocholesterolemic and mildly hypercholesterolemic participants were observed with PS consumption [27, 35, 36, 45, 46]. Reductions in plasma fat soluble compounds concentrations found in these interventions might be because of their lower absorption or due to reduced lipoprotein carriers and serum cholesterol concentrations. However, when concentrations of these fat soluble compounds were corrected for plasma lipid levels, only carotene concentrations were shown to be reduced in some trials [27, 35]. In the current intervention, no such reductions occurred in any of the measured plasma fat soluble compound levels across intervention groups suggesting that other components of the portfolio diet might have compensated for the effect of PS on fat soluble vitamins. Vitamins A and E are essential to maintain normal health and immune system and their deficiency lead to chronic diseases [28, 31]. Consumption of portfolio diet did not affect the plasma concentration of these vitamins which indicates the safety with the portfolio diet with absorption of fat soluble compounds.

Results from current intervention showed no changes across the three dietary groups in plasma concentrations of tocopherols, retinoids and carotenoids, except for a decrease in β-carotene with portfolio groups compared to the control diet group. However, the cholesterol adjusted β-carotene concentrations in plasma showed no differences across all the groups. These findings indicate that consumption of a portfolio diet reduces blood cholesterol but does not affect the absorption of fat soluble vitamins. No changes in cholesterol adjusted fat soluble vitamins concentrations with portfolio diet consumption also indicate that decreased plasma carotenoid concentrations reflect reductions in carrier lipoproteins, especially LDL-C. Results of earlier studies are variable ranging from no change in any of the fat soluble compounds in serum [39, 41, 47, 48] to considerable changes [38] or differences only in β-carotene [49–51]. The lack of consistency in results might be due to different background diets during the intervention and eating habits of the participants. In most of the interventions, diets are not strictly controlled and supervised by study investigators. In contrast, well-controlled human trials with strictly controlled diets have shown no significant differences in concentrations of circulating fat soluble compounds [40, 48].

In the current trial, participants in intensive and routine portfolio diet groups consumed significantly higher amounts of vitamin E compared to participants consuming the control diet, which might also be a reason for not finding any reduction in plasma vitamin E concentrations after consuming portfolio diet. The amount of vitamin A consumption was not significantly different with portfolio dietary groups compared to respective baselines. However, dietary intake of vitamin A by participants in control group was significantly higher than that of the portfolio group which could have contributed to the increase observed in plasma vitamin A concentrations after the control diet intake, compared with the portfolio dietary groups. Several investigators recommended consumption of carotenoid rich foods including fruits and vegetables along with PS consumption to prevent reductions in fat soluble compound levels [12, 51, 52]. PS did not affect the β-carotene absorption in participants with mild hypercholesterolemia when consumed PS esterified to fish oil, but not to sunflower oil [47]. Earlier studies have also shown that PS consumption when distributed over the day results in an optimal cholesterol lowering effect rather than a single large dose [6, 53]. Hence, the background diet and feeding strategy play important roles in terms of effects of PS consumption on plasma concentrations of fat soluble compounds.

Plasma concentrations of retinol and tocopherols were consistent with the ranges seen in our previous publications [47, 48, 54] as well as Sowell et al. [55]. Although the plasma carotenoid concentrations in the current study appear to be slightly higher than values observed by other researchers [55], they were in the same range as found earlier [48]. Plasma samples could be detected with not only the compounds of interest for measurement in the study but also other compounds such as alpha-cryptoxanthin, cis-beta-carotene (13-cis), cislycopene (at least three isomers), cis-lutein/zeaxanthin etc. Presence of the compounds might lead to detection of higher concentration of a given compound which also depends on the relative concentrations of the analyte as well as the interferent. Olmedilla et al. measured the plasma retinol, tocopherol and carotenoid concentrations in the Spanish population and compared their concentrations with those of other country’s populations [56]. Results indicated that carotenoid concentrations showed differences from two to five fold in the reported values. Variations might be due to differences between population and seasonality.

Consumption of our PS containing diet resulted in substantial increases in plasma concentrations of both campesterol and β-sitosterol. Previous trials with humans have shown that consumption of 1.8 g/day of PS increases plasma campesterol and β-sitosterol concentrations from 9 to 16.6 and 4.4 to 6.0 μmol/ L respectively [57]. Results from current work were consistent with the previous data where consumption of portfolio diet consisting 2 g/d of PS increased the plasma PS concentrations [58]. Supplementation of a larger dose of 6.6 g/day of PS led to elevations in circulating campesterol and β-sitosterol concentrations from 7.8 to 15.8 and 8.0 to 11.4 μmol/L respectively [49]. In those trials, plasma PS concentrations were considerably lower than those seen in homozygous phytosterolemic patients who presented with levels of 290–966 μmol/L [17, 59].

Some studies have suggested that moderately increased concentrations of PS in plasma could be associated with increased risk of CVD [13, 14]. Glueck et al. and Assmann et al. observed correlations between campesterol or β-sitosterol alone and CVD risk, respectively [13, 14]. However, these trials concluded that an association exists between plasma PS concentrations and CVD risk, but failed to find the correlations with CVD risk independently from plasma cholesterol concentrations which itself is a risk factor for CVD. A number of investigations with different study designs have shown that plasma PS levels have no association with the risk of CVD [20–22, 60–62]. Results of the current investigation showed significant negative correlations between plasma PS concentrations and total and LDL-C, as well as apoB. However, no significant correlation was found between the Framingham risk score and plasma PS concentrations in the present work. In a trial with 2542 middle aged humans, plasma PS concentrations failed to be associated with increased risk of CVD [21]. Similarly, the prospective EPIC-Norfolk population study had failed to observe any differences in PS concentrations in plasma of healthy participants who developed CVD during the 6 year follow up when compared to their case matched controls [20]. In the Longitudinal Aging Study Amsterdam (LASA), with 1242 participants with 65 years of age, moderately increased concentrations of plasma PS were shown to be associated with reduced CVD risk [19]. Based on these findings, increased plasma PS concentrations due to consumption of PS cannot be considered to be associated with elevated CVD risk. It can be ventured that finite merit exists in maintaining circulating PS levels in the upper range of normal to minimize disease risk.

The study has some limitations as follows. Intake of vitamin E levels during the study was higher with portfolio diet compared with control. In addition, vitamin A intake was higher with control compared to the two portfolio diet groups. Due to the complex nature of the dietary intervention, the vitamin intakes were not same across all the groups. Portfolio diet consisted of combination of various food components and hence the changes in lipids, sterols and vitamins cannot be attributed to one single component. The study was a free living study and not metabolically well controlled by providing all the foods to the participants and follow more closer. Hence, the compliance during the study was less than 50% and drop out rate was 22.6%. However, the aim of the study was to determine the effect of the portfolio diet in real world conditions without much metabolic control and so the drop out rates is as expected. Health benefits could be more than found in the study if the diet is followed more strictly.

In conclusion, consumption of a portfolio diet including PS, viscous fibres, soy proteins and nuts for 6 months reduced blood cholesterol levels without affecting fat soluble vitamin levels. Consequently, the portfolio diet consumption not only reduces serum LDL-C, but also counteracts effects of PS in reducing plasma fat soluble vitamins. Hence, consuming portfolio diet could be considered as one of the best options to maintain normal blood lipid levels and reduce risk of CVD without any adverse effects.

## Abbreviations

CVD: Cardiovascular disease
HDL-C: High density lipoprotein cholesterol
LDL-C: Low density lipoprotein cholesterol
PS: Plant sterols.

## References

[1] Jenkins DJ, Kendall CW, Faulkner D, Vidgen E, Trautwein EA, Parker TL, Marchie A, Koumbridis G, Lapsley KG, Josse RG, Leiter LA, Connelly PW (2002). A dietary portfolio approach to cholesterol reduction: combined effects of plant sterols, vegetable proteins, and viscous fibers in hypercholesterolemia. Metabolism.

[2] Jenkins DJ, Kendall CW, Marchie A, Faulkner D, Vidgen E, Lapsley KG, Trautwein EA, Parker TL, Josse RG, Leiter LA, Connelly PW (2003). The effect of combining plant sterols, soy protein, viscous fibers, and almonds in treating hypercholesterolemia. Metabolism.

[3] Jenkins DJ, Kendall CW, Marchie A, Faulkner DA, Wong JM, de Souza R, Emam A, Parker TL, Vidgen E, Lapsley KG, Trautwein EA, Josse RG, Leiter LA, Connelly PW (2003). Effects of a dietary portfolio of cholesterol-lowering foods vs lovastatin on serum lipids and C-reactive protein. JAMA.

[4] Jenkins DJ, Jones PJ, Lamarche B, Kendall CW, Faulkner D, Cermakova L, Gigleux I, Ramprasath V, de Souza R, Ireland C, Patel D, Srichaikul K, Abdulnour S, Bashyam B, Collier C, Hoshizaki S, Josse RG, Leiter LA, Connelly PW, Frohlich J (2011). Effect of a dietary portfolio of cholesterol-lowering foods given at 2 levels of intensity of dietary advice on serum lipids in hyperlipidemia: a randomized controlled trial. JAMA.

[5] Jenkins DJ, Kendall CW, Faulkner DA, Nguyen T, Kemp T, Marchie A, Wong JM, de Souza R, Emam A, Vidgen E, Trautwein EA, Lapsley KG, Holmes C, Josse RG, Leiter LA, Connelly PW, Singer W (2006). Assessment of the longer-term effects of a dietary portfolio of cholesterol-lowering foods in hypercholesterolemia. Am J Clin Nutr.

[6] Abumweis SS, Barake R, Jones PJ (2008). Plant sterols/stanols as cholesterol lowering agents: a meta-analysis of randomized controlled trials. Food Nutr Res.

[7] Micallef MA, Garg ML (2008). The lipid-lowering effects of phytosterols and (n-3) polyunsaturated fatty acids are synergistic and complementary in hyperlipidemic men and women. J Nutr.

[8] Shrestha S, Volek JS, Udani J, Wood RJ, Greene CM, Aggarwal D, Contois JH, Kavoussi B, Fernandez ML (2006). A combination therapy including psyllium and plant sterols lowers LDL cholesterol by modifying lipoprotein metabolism in hypercholesterolemic individuals. J Nutr.

[9] Theuwissen E, Mensink RP (2007). Simultaneous intake of beta-glucan and plant stanol esters affects lipid metabolism in slightly hypercholesterolemic subjects. J Nutr.

[10] Blair SN, Capuzzi DM, Gottlieb SO, Nguyen T, Morgan JM, Cater NB (2000). Incremental reduction of serum total cholesterol and low-density lipoprotein cholesterol with the addition of plant stanol ester-containing spread to statin therapy. Am J Cardiol.

[11] De Jong A, Plat J, Bast A, Godschalk RW, Basu S, Mensink RP (2008). Effects of plant sterol and stanol ester consumption on lipid metabolism, antioxidant status and markers of oxidative stress, endothelial function and low-grade inflammation in patients on current statin treatment. Eur J Clin Nutr.

[12] Woyengo TA, Ramprasath VR, Jones PJ (2009). Anticancer effects of phytosterols. Eur J Clin Nutr.

[13] Assmann G, Cullen P, Erbey J, Ramey DR, Kannenberg F, Schulte H (2006). Plasma sitosterol elevations are associated with an increased incidence of coronary events in men: results of a nested case–control analysis of the Prospective Cardiovascular Munster (PROCAM) study. Nutr Metab Cardiovasc Dis.

[14] Glueck CJ, Speirs J, Tracy T, Streicher P, Illig E, Vandegrift J (1991). Relationships of serum plant sterols (phytosterols) and cholesterol in 595 hypercholesterolemic subjects, and familial aggregation of phytosterols, cholesterol, and premature coronary heart disease in hyperphytosterolemic probands and their first-degree relatives. Metabolism.

[15] Miettinen TA, Railo M, Lepantalo M, Gylling H (2005). Plant sterols in serum and in atherosclerotic plaques of patients undergoing carotid endarterectomy. J Am Coll Cardiol.

[16] Rajaratnam RA, Gylling H, Miettinen TA (2000). Independent association of serum squalene and noncholesterol sterols with coronary artery disease in postmenopausal women. J Am Coll Cardiol.

[17] Sudhop T, von Bergmann K (2004). Sitosterolemia–a rare disease. Are elevated plant sterols an additional risk factor?. Z Kardiol.

[18] Chan YM, Varady KA, Lin Y, Trautwein E, Mensink RP, Plat J, Jones PJ (2006). Plasma concentrations of plant sterols: physiology and relationship with coronary heart disease. Nutr Rev.

[19] Fassbender K, Lutjohann D, Dik MG, Bremmer M, Konig J, Walter S, Liu Y, Letiembre M, von Bergmann K, Jonker C (2008). Moderately elevated plant sterol levels are associated with reduced cardiovascular risk–the LASA study. Atherosclerosis.

[20] Pinedo S, Vissers MN, von Bergmann K, Elharchaoui K, Lutjohann D, Luben R, Wareham NJ, Kastelein JJ, Khaw KT, Boekholdt SM (2007). Plasma levels of plant sterols and the risk of coronary artery disease: the prospective EPIC-Norfolk Population Study. J Lipid Res.

[21] Wilund KR, Yu L, Xu F, Vega GL, Grundy SM, Cohen JC, Hobbs HH (2004). No association between plasma levels of plant sterols and atherosclerosis in mice and men. Arterioscler Thromb Vasc Biol.

[22] Windler E, Zyriax BC, Kuipers F, Linseisen J, Boeing H (2009). Association of plasma phytosterol concentrations with incident coronary heart disease Data from the CORA study, a case–control study of coronary artery disease in women. Atherosclerosis.

[23] Jones PJ, Raeini-Sarjaz M, Ntanios FY, Vanstone CA, Feng JY, Parsons WE (2000). Modulation of plasma lipid levels and cholesterol kinetics by phytosterol versus phytostanol esters. J Lipid Res.

[24] Carroll KK (1991). Review of clinical studies on cholesterol-lowering response to soy protein. J Am Diet Assoc.

[25] Jenkins DJA, Wolever TMS, Rao AV, Hegele RA, Mitchell SJ, Ransom TPP, Boctor DL, Spadafora PJ, Jenkins AL, Mehling C, Relle LK, Connelly PW, Story JA, Furumoto EJ, Corey P, Wursch P (1993). Effect on blood-lipids of very high intakes of fiber in diets low in saturated fat and cholesterol. New Engl J Med.

[26] Jenkins DJ, Kendall CW, Marchie A, Parker TL, Connelly PW, Qian W, Haight JS, Faulkner D, Vidgen E, Lapsley KG, Spiller GA (2002). Dose response of almonds on coronary heart disease risk factors: blood lipids, oxidized low-density lipoproteins, lipoprotein(a), homocysteine, and pulmonary nitric oxide: a randomized, controlled, crossover trial. Circulation.

[27] Hendriks HF, Weststrate JA, van Vliet T, Meijer GW (1999). Spreads enriched with three different levels of vegetable oil sterols and the degree of cholesterol lowering in normocholesterolaemic and mildly hypercholesterolaemic subjects. Eur J Clin Nutr.

[28] Tanumihardjo SA (2011). Vitamin A: biomarkers of nutrition for development. Am J Clin Nutr.

[29] Mayo-Wilson E, Imdad A, Herzer K, Yakoob MY, Bhutta ZA (2011). Vitamin A supplements for preventing mortality, illness, and blindness in children aged under 5: systematic review and meta-analysis. BMJ.

[30] Brigelius-Flohe R, Traber MG (1999). Vitamin E: function and metabolism. FASEB J.

[31] Schneider C (2005). Chemistry and biology of vitamin E. Mol Nutr Food Res.

[32] Traber MG, Stevens JF (2011). Vitamins C and E: beneficial effects from a mechanistic perspective. Free Radic Biol Med.

[33] Berger A, Jones PJ, Abumweis SS (2004). Plant sterols: factors affecting their efficacy and safety as functional food ingredients. Lipids Health Dis.

[34] de Jong A, Plat J, Mensink RP (2003). Metabolic effects of plant sterols and stanols (Review). J Nutr Biochem.

[35] Katan MB, Grundy SM, Jones P, Law M, Miettinen T, Paoletti R (2003). Efficacy and safety of plant stanols and sterols in the management of blood cholesterol levels. Mayo Clinic Proc Mayo Clin.

[36] Law M (2000). Plant sterol and stanol margarines and health. BMJ.

[37] Plat J, Mensink RP (2005). Plant stanol and sterol esters in the control of blood cholesterol levels: mechanism and safety aspects. Am J Cardiol.

[38] Richelle M, Enslen M, Hager C, Groux M, Tavazzi I, Godin JP, Berger A, Metairon S, Quaile S, Piguet-Welsch C, Sagalowicz L, Green H, Fay LB (2004). Both free and esterified plant sterols reduce cholesterol absorption and the bioavailability of beta-carotene and alpha-tocopherol in normocholesterolemic humans. Am J Clin Nutr.

[39] Korpela R, Tuomilehto J, Hogstrom P, Seppo L, Piironen V, Salo-Vaananen P, Toivo J, Lamberg-Allardt C, Karkkainen M, Outila T, Sundvall J, Vilkkilä S, Tikkanen MJ (2006). Safety aspects and cholesterol-lowering efficacy of low fat dairy products containing plant sterols. Eur J Clin Nutr.

[40] Rudkowska I, AbuMweis SS, Nicolle C, Jones PJ (2008). Cholesterol-lowering efficacy of plant sterols in low-fat yogurt consumed as a snack or with a meal. J Am Coll Nutr.

[41] Thomsen AB, Hansen HB, Christiansen C, Green H, Berger A (2004). Effect of free plant sterols in low-fat milk on serum lipid profile in hypercholesterolemic subjects. Eur J Clin Nutr.

[42] Volpe R, Niittynen L, Korpela R, Sirtori C, Bucci A, Fraone N, Pazzucconi F (2001). Effects of yoghurt enriched with plant sterols on serum lipids in patients with moderate hypercholesterolaemia. Br J Nutr.

[43] Gueguen S, Herbeth B, Siest G, Leroy P (2002). An isocratic liquid chromatographic method with diode-array detection for the simultaneous determination of alpha-tocopherol, retinol, and five carotenoids in human serum. J Chromatogr Sci.

[44] Varady KA, Ebine N, Vanstone CA, Parsons WE, Jones PJ (2004). Plant sterols and endurance training combine to favorably alter plasma lipid profiles in previously sedentary hypercholesterolemic adults after 8 wk. Am J Clin Nutr.

[45] Weststrate JA, Meijer GW (1998). Plant sterol-enriched margarines and reduction of plasma total- and LDL-cholesterol concentrations in normocholesterolaemic and mildly hypercholesterolaemic subjects. Eur J Clin Nutr.

[46] Jones PJ, AbuMweis SS (2009). Phytosterols as functional food ingredients: linkages to cardiovascular disease and cancer. Curr Opin Clin Nutr Metab Care.

[47] Jones PJ, Demonty I, Chan YM, Herzog Y, Pelled D (2007). Fish-oil esters of plant sterols differ from vegetable-oil sterol esters in triglycerides lowering, carotenoid bioavailability and impact on plasminogen activator inhibitor-1 (PAI-1) concentrations in hypercholesterolemic subjects. Lipids Health Dis.

[48] Raeini-Sarjaz M, Ntanios FY, Vanstone CA, Jones PJ (2002). No changes in serum fat-soluble vitamin and carotenoid concentrations with the intake of plant sterol/stanol esters in the context of a controlled diet. Metabolism.

[49] Clifton PM, Noakes M, Ross D, Fassoulakis A, Cehun M, Nestel P (2004). High dietary intake of phytosterol esters decreases carotenoids and increases plasma plant sterol levels with no additional cholesterol lowering. J Lipid Res.

[50] Mensink RP, Ebbing S, Lindhout M, Plat J, van Heugten MM (2002). Effects of plant stanol esters supplied in low-fat yoghurt on serum lipids and lipoproteins, non-cholesterol sterols and fat soluble antioxidant concentrations. Atherosclerosis.

[51] Noakes M, Clifton P, Ntanios F, Shrapnel W, Record I, McInerney J (2002). An increase in dietary carotenoids when consuming plant sterols or stanols is effective in maintaining plasma carotenoid concentrations. Am J Clin Nutr.

[52] QuIlez J, GarcIa-Lorda P, Salas-Salvado J (2003). Potential uses and benefits of phytosterols in diet: present situation and future directions. Clin Nutr.

[53] AbuMweis SS, Vanstone CA, Lichtenstein AH, Jones PJ (2009). Plant sterol consumption frequency affects plasma lipid levels and cholesterol kinetics in humans. Eur J Clin Nutr.

[54] Demonty I, Chan YM, Pelled D, Jones PJ (2006). Fish-oil esters of plant sterols improve the lipid profile of dyslipidemic subjects more than do fish-oil or sunflower oil esters of plant sterols. Am J Clin Nutr.

[55] Sowell AL, Huff DL, Yeager PR, Caudill SP, Gunter EW (1994). Retinol, alpha-tocopherol, lutein/zeaxanthin, beta-cryptoxanthin, lycopene, alpha-carotene, trans-beta-carotene, and four retinyl esters in serum determined simultaneously by reversed-phase HPLC with multiwavelength detection. Clin Chem.

[56] Olmedilla B, Granado F, Gil-Martinez E, Blanco I, Rojas-Hidalgo E (1997). Reference values for retinol, tocopherol, and main carotenoids in serum of control and insulin-dependent diabetic Spanish subjects. Clin Chem.

[57] Mussner MJ, Parhofer KG, Von Bergmann K, Schwandt P, Broedl U, Otto C (2002). Effects of phytosterol ester-enriched margarine on plasma lipoproteins in mild to moderate hypercholesterolemia are related to basal cholesterol and fat intake. Metabolism.

[58] Jones PJ, Raeini-Sarjaz M, Jenkins DJ, Kendall CW, Vidgen E, Trautwein EA, Lapsley KG, Marchie A, Cunnane SC, Connelly PW (2005). Effects of a diet high in plant sterols, vegetable proteins, and viscous fibers (dietary portfolio) on circulating sterol levels and red cell fragility in hypercholesterolemic subjects. Lipids.

[59] Salen G, Shefer S, Nguyen L, Ness GC, Tint GS, Shore V (1992). Sitosterolemia. J Lipid Res.

[60] Sudhop T, Gottwald BM, von Bergmann K (2002). Serum plant sterols as a potential risk factor for coronary heart disease. Metabolism.

[61] Silbernagel G, Genser B, Nestel P, Marz W (2013). Plant sterols and atherosclerosis. Curr Opin Lipidol.

[62] Genser B, Silbernagel G, De Backer G, Bruckert E, Carmena R, Chapman MJ, Deanfield J, Descamps OS, Rietzschel ER, Dias KC, Marz W (2012). Plant sterols and cardiovascular disease: a systematic review and meta-analysis. Eur Heart J.

